# Mirror Effect of Parvalbumin and Connexin 43 Expression in the Acute and Subacute Phases After Penetrating Traumatic Brain Injury Reveals a Non-Canonical Interaction

**DOI:** 10.3390/molecules31061018

**Published:** 2026-03-18

**Authors:** Oleg Kit, Evgeniya Kirichenko, Stanislav Bachurin, Rozaliia Nabiullina, Chizaram Nwosu, Pavel Sakun, Stanislav Rodkin

**Affiliations:** 1 Federal State Budgetary Institution “National Medical Research Center of Oncology” Russian Federation, 14th Line St., 63, Rostov-on-Don 344037, Russia; 2 Research Laboratory “Medical Digital Images Based on the Basic Model”, Department of Bioengineering, Faculty of Bioengineering and Veterinary Medicine, Don State Technical University, Rostov-on-Don 344000, Russia

**Keywords:** traumatic brain injury, parvalbumin, connexin 43, calcium homeostasis, neuroglial interaction, excitotoxicity, NeuN, secondary brain injury, molecular dynamics modeling

## Abstract

Traumatic brain injury (TBI) initiates a cascade of molecular and cellular reactions leading to long-term disturbances of neuronal and glial homeostasis. One of the key mechanisms of secondary injury is a pathological increase in intracellular Ca^2+^ concentration. Parvalbumin (PV) plays an important role in the regulation of Ca^2+^ homeostasis in neurons. In turn, connexin 43 (Cx43) is the principal protein of astrocytic gap junctions (GJs), which ensure neuroglial communication. The spatiotemporal changes in these proteins and the mechanisms of their interaction after TBI remain insufficiently studied. In the present study, a comprehensive analysis of the expression, localization, and spatial organization of PV and Cx43 in the cerebral cortex following TBI was performed. In intact tissue, PV was localized predominantly in neurons, whereas Cx43 formed typical punctate structures of astrocytic GJs. Twenty-four hours after TBI, a sharp activation of PV with pronounced nuclear translocation was observed against the background of a catastrophic decrease in Cx43 expression, accompanied by a reduction in the number of NeuN^+^ neurons and signs of apoptosis. However, after 7 days, a mirror-opposite effect was detected, characterized by decreased PV expression and increased Cx43 levels with its aggregation into cluster-like structures, as well as partial restoration of NeuN immunoreactivity. In addition, molecular dynamics simulations demonstrated that the stability of the PV–Cx43 complex is determined by the presence of Ca^2+^ and physiological pH, whereas acidosis and Ca^2+^ overload destabilize their interaction. Taken together, these results reveal a phase-dependent mirror-opposite pattern of PV and Cx43 expression and localization and emphasize the key role of Ca^2+^- and pH-dependent neuroglial interactions in TBI.

## 1. Introduction

Traumatic brain injury (TBI) remains one of the leading causes of traumatic damage to the central nervous system (CNS), often resulting in disability or death [1,2]. Despite extensive research in this field, there are still no selective neuroprotective agents that have demonstrated consistent efficacy in clinical trials by effectively protecting injured neurons and glial cells after trauma [3]. Therefore, the search for new molecular targets for selective neuroprotective therapy remains highly relevant.

It is well established that a critical link in the chain of pathomorphological processes induced by TBI is the disruption of Ca^2+^ homeostasis in the CNS, leading to a neurotoxic increase in intracellular Ca^2+^ concentration through mechanisms involving NMDA receptor activation and other signaling pathways [4,5,6]. As a result, Ca^2+^-mediated excitotoxicity develops, triggering cascades of pathological processes associated with depletion of energy resources, accumulation of toxic metabolites, and the emergence of widespread intracellular disturbances in neurons that ultimately induce cell death [7]. In these complex processes, parvalbumin (PV) a small Ca^2+^-binding protein predominantly localized in fast-spiking GABAergic interneurons (PV-Ins) plays an active role. These neurons provide inhibitory control in the cortex and hippocampus and are crucial for neuronal synchronization and the generation of gamma rhythms [8]. The loss or functional impairment of these cells following TBI leads to severe disturbances in inhibitory control, excessive neuronal depolarization, and an increased risk of seizures and other CNS disorders [9,10].

PV-INs are involved in neuroglial interactions in which astrocytes play a central role by forming extensive networks of intercellular communication via gap junctions (GJs). Astrocytic GJs are, in turn, critical for the survival of neurons and glial cells after TBI, and their main structural component is connexin 43 (Cx43), a small protein containing four transmembrane domains, two extracellular loops, and cytoplasmic N- and C-terminal regions. Cx43 forms hemichannels composed of six Cx43 monomers. The docking of two hemichannels forms a functional GJ, thereby supporting neuronal homeostasis through the transport of ions and metabolites and even certain intracellular organelles, such as mitochondria [11,12,13]. Following TBI, alterations in the activity of Cx43-associated channels, as well as changes in Cx43 expression and phosphorylation, have been observed and are associated either with the expansion of secondary injury or with the activation of neuroprotective mechanisms [14]. In addition, Cx43 expression has been reported in certain neuronal populations [15].

To date, it is known that complex mechanisms of interaction exist between astrocytes and PV-INs [16], which may also be mediated through GJs. PV interneurons form an intricate network of electrical synapses that regulate the activity and synchronization of entire ensembles of cortical neurons. This electrically coupled network is mediated by GJs in which Cx43 also participates, providing an important mechanism for PV-IN synchronization [17]. Moreover, GABAergic interneurons have been shown to induce Ca^2+^ responses in astrocytes, thereby initiating glial signaling cascades associated with neuronal homeostasis [16,18]. Such neuroglial interactions, mediated in part by GJs, represent complex mechanisms that are highly sensitive to various extracellular disturbances, including those caused by traumatic injury to the nervous system.

Importantly, PV-positive interneurons are ensheathed by perisomatic astrocytic processes enriched in Cx43-containing GJs, forming shared ionic and signaling microdomains [19]. Moreover, Cx43 expression is not restricted to astrocytes but is also detected in neuronal populations, with up to ~40% of PV-positive interneurons reported to express this connexin [15]. Because GJs mediate metabolic coupling and synchronization of GABAergic neuronal networks, such spatial organization implies close proximity between PV-containing cytoplasm and intracellular domains of Cx43. These features suggest that transient Ca^2+^- and pH-dependent molecular interactions between the two proteins may occur under pathological conditions, although the existence of a stable structural complex in vivo has not been demonstrated. Therefore, the spatiotemporal changes in PV and Cx43 following TBI are of both fundamental and practical interest in the search for new neuroprotective mechanisms. However, the spatiotemporal co-expression and localization of Cx43 and PV in neurons and glial cells have not been previously investigated and are of particular interest under conditions of trauma-induced neuronal stress.

In the context of studying such subtle cellular interactions, methods for objective assessment of morphological and molecular changes play a crucial role. Modern biomedical research increasingly requires quantitative analysis of images of complex biological structures, involving the identification, localization, and quantitative evaluation of cellular or subcellular components [20]. However, this process still largely depends on manual work and the expertise of the researcher and is associated with subjectivity and variability in data interpretation. The use of automated image analysis systems allows for a significant acceleration of image processing, improved measurement accuracy, and minimization of errors [21], which is especially important when studying dynamic and heterogeneous structures such as GJs.

In our recent study, we demonstrated that TBI is accompanied by phase-dependent changes in Cx43 expression, associated with apoptotic neuronal degradation in the acute period and pronounced reactive astrogliosis in the subacute phase. In addition, a close relationship was identified between Cx43 levels, systemic immune shifts, and pH-dependent conformational reorganization of Cx43, potentially determining its neurotoxic effects in the post-traumatic period [22]. We also performed a detailed analysis of p53-dependent cell death by developing an IT-based algorithm for the analysis of fragmented cell nuclei in nervous tissue following TBI [23].

In the present study, we comprehensively investigated the expression and subcellular localization of PV in combination with Cx43 in a mouse model of parietal TBI using high-resolution laser scanning confocal microscopy, optical immunohistochemistry, and neural network–based algorithms for quantitative assessment of aggregated Cx43 clusters in microscopic images. Particular attention was paid to the spatiotemporal redistribution of signals within neuron–astrocyte microdomains and to distinguishing intracellular, perisynaptic, and extracellular localization patterns after injury. In parallel, we performed in silico modeling of potential PV–Cx43 interaction under conditions of TBI-induced acidosis and calcium overload. This modeling was intended to evaluate the physicochemical feasibility of transient interaction in a shared ionic environment rather than to demonstrate the existence of a stable protein complex in vivo. Thus, we propose a non-classical mechanistic model in which Ca^2+^- and pH-dependent coupling between neuronal calcium buffering and astrocytic GJ remodeling may contribute to the coordinated post-traumatic response of neural tissue.

## 2. Results

### 2.1. Spatiotemporal Comparison of PV and Cx43 Expression

**Figure 1:**
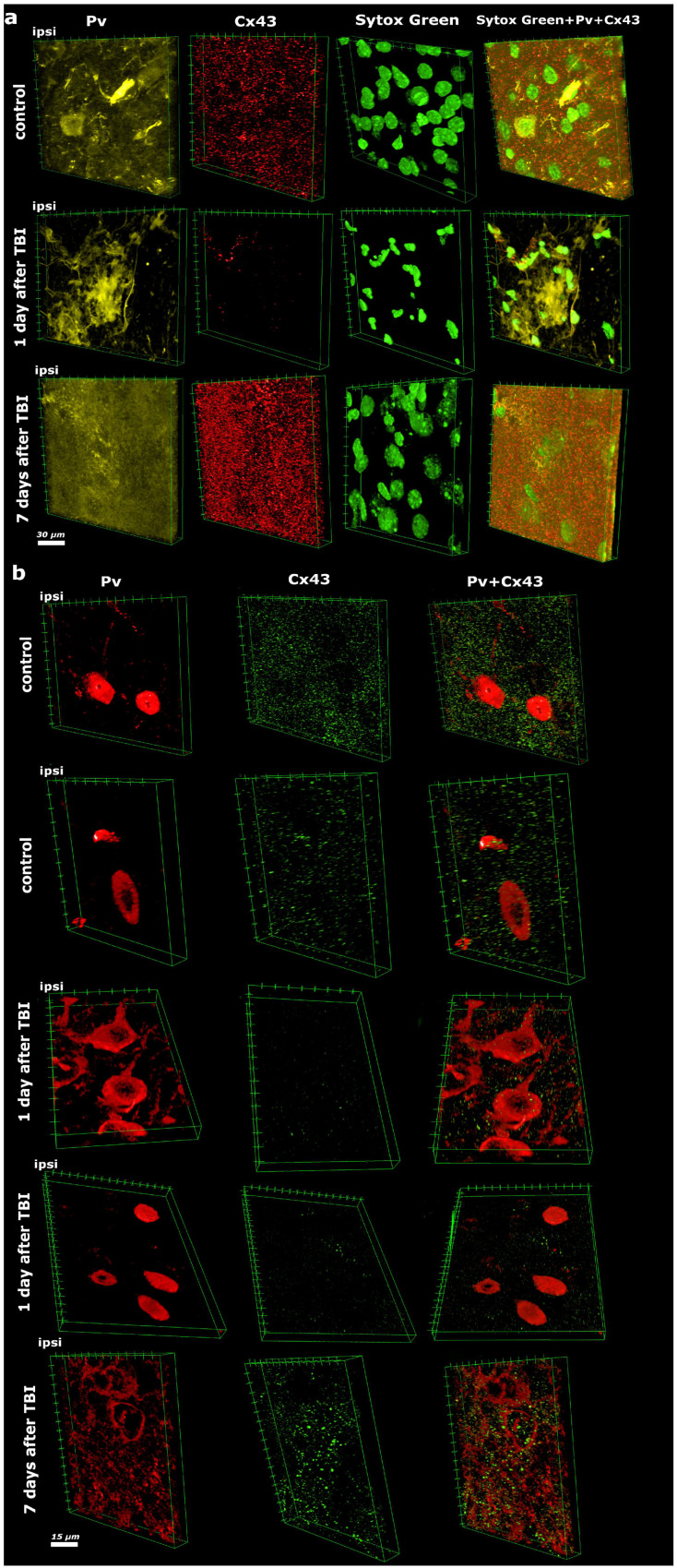
Confocal laser scanning microscopy illustrating PV and Cx43 expression in the ipsilateral cerebral hemispheres of control and experimental animals at 1 and 7 days after TBI in the parietal cortex. (**a**)—Dark yellow fluorescence indicates PV immunolabeling, red corresponds to Cx43, and green denotes nuclear staining with Sytox Green. Colocalization of these markers is visualized by signal overlay and 3D reconstruction in the final images (Sytox Green, PV, and Cx43). Scale bar: 30 μm. (**b**)—Red fluorescence indicates PV immunolabeling, while green corresponds to Cx43. Colocalization is visualized by signal overlay and 3D reconstruction in the final images (PV and Cx43). Scale bar: 15 μm.

Three-dimensional analysis of the spatial distribution of PV and Cx43 in the brain revealed a pronounced, mirror-opposite pattern of these two proteins during the acute and subacute post-traumatic periods after TBI (Figure 1). Observations were obtained from the ipsilateral perilesional parietal cortex adjacent to the impact site (excluding the necrotic core) and from the corresponding region of the contralateral hemisphere used as an internal control. Unlike single optical sections, volumetric reconstruction of the Z-stacks allowed discrimination between true intracellular localization and signal overlap caused by projection of structures located at different depths of the tissue, which is particularly critical in densely packed cortical neuropil.

In the ipsilateral hemisphere of the control group, which was not subjected to direct mechanical impact, immunofluorescent staining for PV combined with confocal laser scanning microscopy demonstrated a baseline level of expression typical of intact nervous tissue. PV was detected predominantly in the cytoplasm and, in some neurons, within the nuclei. Cx43 expression in the intact hemisphere corresponded to physiological norms and appeared as punctate structures uniformly distributed throughout the neuropil, reflecting normal functioning of astrocytic gap junctions (Figure 1). Three-dimensional rendering confirmed that these puncta were localized along astrocytic processes surrounding neuronal somata rather than randomly overlapping with neuronal profiles, excluding false colocalization that may occur in two-dimensional projections.

At 24 h after TBI, a sharp activation of PV expression was observed in the ipsilateral hemisphere, markedly exceeding baseline levels. Three-dimensional reconstruction of confocal Z-stacks revealed a pronounced increase in PV content within the cytoplasm, neuronal processes, and nuclei. Orthogonal views verified that nuclear PV accumulation represented true intranuclear translocation rather than perinuclear signal overlay from adjacent planes. Simultaneously, Cx43 expression catastrophically declined to nearly undetectable levels. The typical punctate Cx43 structures disappeared, indicating rapid disruption of astrocytic gap junctions during the acute phase of TBI. In addition, severe destructive changes were observed, including nuclear fragmentation, pyknotic shrinkage, gross alterations in nuclear morphology, and a significant decrease in nuclear density within the lesion area (Figure 1), reflecting extensive neuronal and glial cell death during the acute phase of injury.

Seven days after TBI, the expression pattern of both proteins exhibited a clearly opposite trend. PV levels in the ipsilateral hemisphere sharply decreased and became markedly lower than those in the intact hemisphere as well as in the injured cortex at 24 h post-trauma. Only single neurons retained a weak cytoplasmic PV signal, while nuclear localization of PV completely disappeared, indicating the transient nature of TBI-induced PV expression. At the same time, a pronounced fine-granular PV-reactive dispersion was detected in the extracellular space, reaching maximal intensity at 7 days after TBI. Three-dimensional segmentation demonstrated that a substantial fraction of PV-positive signal was located outside cellular boundaries within perineuronal and interstitial spaces, a feature that could not be reliably distinguished from cytoplasmic staining in two-dimensional images. Three-dimensional analysis demonstrated diffuse distribution of PV-positive granules in the perineuronal space and neuropil (Figure 1), which may indicate release of PV from damaged neurons and its accumulation in the extracellular milieu during the late post-traumatic phase.

In contrast, Cx43 expression at 7 days after injury was markedly increased, substantially exceeding baseline levels observed in the intact hemisphere. Three-dimensional analysis revealed not only a quantitative increase in Cx43 but also a qualitative alteration in its spatial organization, manifested by the formation of large protein aggregates (Figure 1). Volumetric reconstruction further showed that these aggregates formed elongated multilayer structures extending across several optical planes, consistent with remodeling of astrocytic GJ plaques rather than simple enlargement of individual puncta. Destructive nuclear changes persisted; however, nuclear density within the lesion area began to increase, likely due to infiltration of inflammatory cells and proliferation of reactive glia.

### 2.2. Immunofluorescence Analysis of PV and Cx43 Expression and Localization

Immunofluorescence microscopy combined with quantitative analysis of fluorescence intensity and the M1 colocalization coefficient enabled a detailed assessment of the phase-dependent changes in PV and Cx43 expression and localization, revealing a mirror-opposite pattern of their responses to traumatic injury in the acute and subacute phases after TBI.

**Figure 2:**
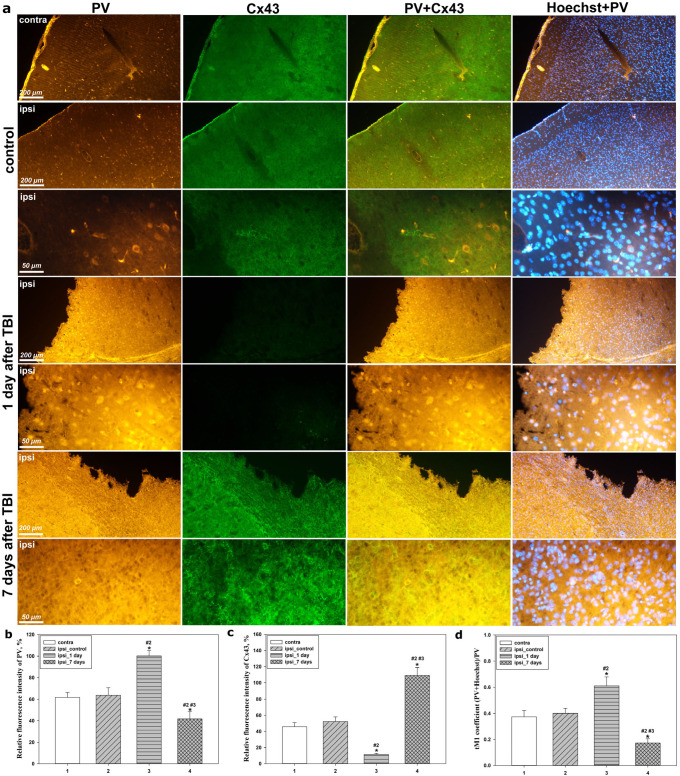
Immunofluorescence analysis of PV and Cx43 expression in the contralateral and ipsilateral cerebral cortex of control and experimental animals at 24 h and 7 days after TBI in the parietal cortex. (**a**)—Immunofluorescence microscopy showing PV expression (yellow fluorescence) and Cx43 expression (green fluorescence), as well as colocalization with Hoechst 33342 (blue fluorescence) and PV. Signal overlays are shown in the PV + Cx43 and Hoechst + PV panels. Scale bars: 50 μm and 200 μm. (**b**)—Mean fluorescence intensity of PV in the contralateral and ipsilateral cortex at 24 h and 7 days after TBI. (**c**)—Mean fluorescence intensity of Cx43 in the contralateral and ipsilateral cortex at 24 h and 7 days after TBI. (**d**)—M1 colocalization coefficient between PV and Hoechst signals in the cerebral cortex at 24 h and 7 days after TBI. Numbers below the bars correspond to: 1—contralateral cortex; 2—control ipsilateral cortex; 3—ipsilateral cortex at 1 day after TBI; 4—ipsilateral cortex at 7 days after TBI. * *p* < 0.05 indicates a significant difference between ipsilateral and contralateral cortex; # *p* < 0.05 indicates a significant difference between experimental and control ipsilateral cortex. Statistical significance is indicated for the respective groups (#2, #3). Data are presented as mean ± SEM (*n* = 6). Statistical analysis was performed using ANOVA followed by Tukey’s post hoc test.

In the contralateral hemisphere, as well as in the ipsilateral cortex of sham-operated animals, the levels of PV and Cx43 remained stable and comparable across groups (Figure 2a). In these groups, PV was localized predominantly in the cytoplasm and neuronal processes, displaying a uniform distribution and a characteristic moderate fluorescence intensity (Figure 2a,b). PV-positive (PV^+^) cells with nuclear localization of this protein were also detected, as confirmed by the M1 colocalization coefficient values (Figure 2d). In turn, Cx43 expression in control animals appeared as diffusely distributed small punctate structures (Figure 2a), corresponding to the normal morphology of astrocytic GJ.

At 24 h after TBI, substantial changes in PV expression and localization were observed. The fluorescence intensity of PV in the ipsilateral cortex increased significantly by 56% compared with control values (*p* < 0.05) (Figure 2b). Simultaneously, the M1 colocalization coefficient increased by 50% (*p* < 0.05), reflecting a pronounced shift in PV toward nuclear localization (Figure 2d). Visually, this was manifested as brighter, denser, and more concentrated nuclear staining, indicating an early reorganization of the functional state of PV^+^ neurons. In addition, as early as 24 h after TBI, an increase in diffuse background PV signal was detected in the neuropil of the ipsilateral cortex, which may indicate initial accumulation of PV-immunoreactive material in the extracellular space as a result of cell death. Against this background, Cx43 expression exhibited the opposite trend: its level decreased more than fourfold relative to control (*p* < 0.05), accompanied by the loss of characteristic punctate structures (Figure 2c). These changes indicate rapid disruption of the astrocytic network during the acute phase of the post-traumatic period.

At 7 days after TBI, pronounced changes in PV expression dynamics were observed (Figure 2a). PV fluorescence intensity significantly decreased by 34% relative to control values (*p* < 0.05) and was 2.4-fold lower than the level observed at 24 h after TBI (*p* < 0.05) (Figure 2b). The M1 coefficient also markedly decreased by 2.3-fold relative to control and by 3.5-fold (*p* < 0.05) relative to the 24 h injury group (Figure 2d), indicating reduced nuclear localization and a return of the protein to predominantly cytoplasmic distribution. At this stage, the diffuse PV signal in the extracellular space persisted and was more pronounced than in the control group, manifesting as a general increase in background fluorescence within the neuropil.

In contrast to PV, Cx43 expression at this time point increased significantly. Its fluorescence intensity exceeded control levels by 2.2-fold (*p* < 0.05) and was almost 10-fold higher than that observed at 24 h after TBI (*p* < 0.05) (Figure 2c). The signal appeared as large bright aggregates and elongated linear clusters (Figure 2a), reflecting active structural and functional reorganization of astrocytic GJs during the subacute stage of the post-traumatic period.

**Figure 3:**
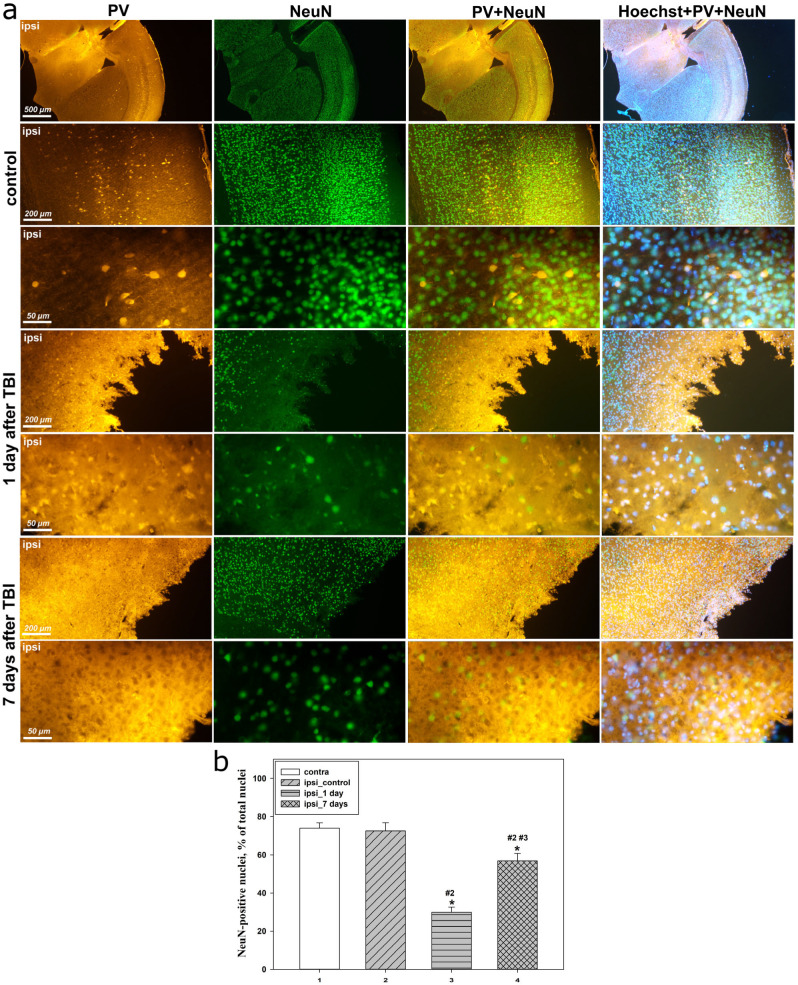
Immunofluorescence analysis of PV and NeuN expression in the contralateral and ipsilateral cerebral cortex of control and experimental animals at 24 h and 7 days after TBI in the parietal cortex. (**a**)—Immunofluorescence microscopy showing PV expression (yellow fluorescence) and NeuN expression (green fluorescence), as well as their colocalization with Hoechst 33342 (blue fluorescence). Signal overlays are shown in the PV + NeuN and Hoechst + PV + NeuN panels. Scale bars: 50 μm, 200 μm, and 500 μm. (**b**)—Proportion of NeuN-positive nuclei expressed as a percentage of the total number of Hoechst-positive nuclei in the contralateral and ipsilateral cortex at 24 h and 7 days after TBI. Numbers below the bars correspond to: 1—contralateral cortex; 2—control ipsilateral cortex; 3—ipsilateral cortex at 1 day after TBI; 4—ipsilateral cortex at 7 days after TBI. * *p* < 0.05 indicates a significant difference between ipsilateral and contralateral cortex; # *p* < 0.05 indicates a significant difference between experimental and control ipsilateral cortex. Data are presented as mean ± SEM (*n* = 6). Statistical analysis was performed using ANOVA followed by Tukey’s post hoc test.

Additional immunofluorescence analysis using the neuronal marker NeuN demonstrated that PV predominantly colocalized with NeuN-positive (NeuN^+^) cells in all groups (Figure 3a), confirming the neuronal identity of PV-immunoreactive structures.

A distinctive feature of the acute post-traumatic phase was the pronounced accumulation of PV within the nuclei of NeuN^+^ neurons, predominantly in the cortex located in close proximity to the injury focus. In these regions, the most intense and compact nuclear PV staining was observed, whereas with increasing distance from the injury site, nuclear localization became less pronounced and was replaced by predominantly cytoplasmic distribution (Figure 3a).

At later time points after TBI, nuclear localization of PV in NeuN^+^ neurons was markedly attenuated, accompanied by restoration of predominantly cytoplasmic PV distribution and a decrease in its degree of colocalization with NeuN and Hoechst. These changes correlated with partial recovery of the neuronal phenotype and a reduction in the severity of nuclear morphological abnormalities observed during the acute phase (Figure 3a).

In addition, quantitative analysis of the proportion of NeuN^+^ nuclei relative to the total number of Hoechst-positive nuclei allowed characterization of the phase-dependent changes in neuronal phenotype in the cerebral cortex after TBI.

In the contralateral cortex, as well as in the ipsilateral cortex of control animals, the proportion of NeuN^+^ nuclei remained stable and comparable across groups. However, at 24 h after TBI, the ipsilateral cortex exhibited a sharp and statistically significant decrease in the proportion of NeuN-positive nuclei by 2.5-fold compared with the control ipsilateral cortex (*p* < 0.05) (Figure 3b). These changes were accompanied by pronounced nuclear morphological abnormalities, including chromatin condensation and the appearance of fragmented nuclei characteristic of apoptotic processes (Figure 3a).

At 7 days after TBI, a partial recovery of the proportion of NeuN^+^ nuclei was observed, reaching a 96% increase relative to the 24 h group (*p* < 0.05); however, this parameter remained approximately 23% lower than control levels (*p* < 0.05) (Figure 3b). These changes were accompanied by a reduction in the number of fragmented nuclei and clearer visualization of the NeuN signal (Figure 3a).

### 2.3. Performance of the Computer Vision Model for Assessing Cx43 Aggregation

For automated analysis of immunohistochemical images, a computer vision model based on the DINO-SwinL architecture was trained using the AutoGluon framework (autogluon.multimodal 1.1.1). The detector was trained and applied to individual optical sections extracted from confocal Z-stacks rather than to reconstructed volumetric images. On a held-out test dataset, the model demonstrated high generalization performance, achieving an overall mAP@50 of 78.4% across all object classes. Class-wise analysis of average precision (AP) revealed lower detection accuracy for aggregated structures (72.5%) compared with isolated structures, for which the AP reached 84.3%.

Qualitative error analysis showed that the majority of false-positive and false-negative detections were associated with weakly saturated clusters or closely spaced punctate structures located at the boundary of admissible cases according to the predefined annotation rules. Because the analysis was performed in single optical planes, the model was able to identify small condensed puncta that may not be reliably distinguishable after volumetric projection during 3D rendering.

**Figure 4:**
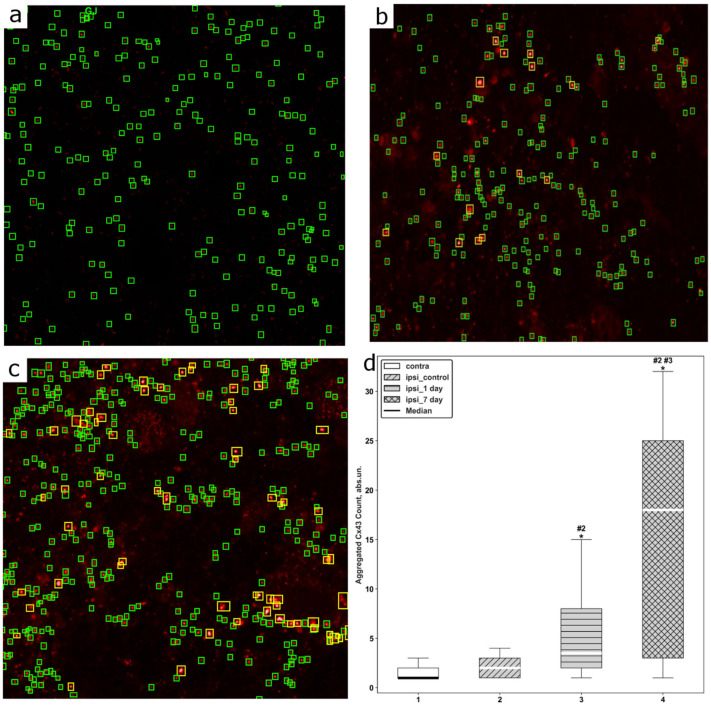
Examples of model predictions for punctate Cx43-associated structures and quantitative analysis of aggregated Cx43. (**a**)—Control hemisphere; (**b**)—injured hemisphere at 1 day after TBI; (**c**)—injured hemisphere at 7 days after TBI. Green bounding boxes indicate individual Cx43-associated structures corresponding to the normal Cx43 distribution, whereas yellow bounding boxes denote Cx43-associated aggregated clusters. (**d**)—Box-and-whisker plots summarizing the distribution of aggregated Cx43 across control and experimental groups. Boxes represent the interquartile range, the horizontal line indicates the median, whiskers denote the 10th–90th percentiles, and outliers are excluded for clarity. * *p* < 0.05 indicates a significant difference between ipsilateral and contralateral cortex; # *p* < 0.05 indicates a significant difference between experimental and control ipsilateral cortex. All data are presented as absolute values of the aggregated number of Cx43 puncta per ROI. Aggregation reflects structural reorganization of Cx43 rather than total protein expression level. Detection was performed on individual optical sections, not 3D reconstructions.

Analysis of Cx43 expression revealed pronounced changes following TBI. Descriptive statistics demonstrated a progressive increase in the number of aggregated Cx43 structures in all experimental groups compared with controls (Figure 4). Notably, at 24 h after TBI many detected aggregates corresponded to small fragmented or condensed puncta observable in single optical sections but poorly distinguishable in volumetric 3D reconstructions. Because the detection algorithm operates on individual Z-stack planes, it is more sensitive to early micro-clusters, whereas 3D rendering primarily visualizes larger aggregates that become evident at later stages. Thus, the apparent discrepancy reflects different detection scales rather than increased overall Cx43 expression.

Assessment of data distributions using the Shapiro–Wilk test confirmed non-normality in all groups (*p* < 0.05), necessitating the use of nonparametric statistical methods. The amount of aggregated Cx43 was analyzed across all groups, revealing a consistent increase in the number of aggregated Cx43 clusters with progression from control conditions to later post-traumatic time points (Figure 4a–c). The Kruskal–Wallis test identified statistically significant differences between groups (H(3) = 61.54; *p* < 0.05). The effect size was large (η^2^ = 0.297), indicating that group membership accounted for approximately 30% of the variability in the degree of Cx43 aggregation.

Post hoc analysis using the Mann–Whitney U test with Benjamini–Hochberg false discovery rate (FDR) correction revealed statistically significant differences between most group comparisons. The only nonsignificant comparison was between the contralateral and ipsilateral hemispheres in the control group (adjusted *p* = 0.206), which was also confirmed using Holm correction.

Additional post hoc analysis with Holm correction demonstrated that, in the ipsilateral injury zone, a statistically significant increase in Cx43 aggregation was already evident at 24 h after TBI compared with the contralateral hemisphere (*p* < 0.05). This trend became more pronounced at 7 days after injury, both in comparison with the contralateral hemisphere (*p* < 0.05) and with the ipsilateral hemisphere at 24 h after TBI (*p* < 0.05) (Figure 4d).

Effect sizes calculated using Cliff’s delta further confirmed the robustness of the observed differences. Specifically, comparison of the contralateral hemisphere of the control group with the ipsilateral cortex at 24 h after TBI yielded an effect size of δ = −0.50, while comparison with the 7-day post-TBI group yielded δ = −0.70. Similarly, comparison of the control group with the ipsilateral cortex at 7 days after TBI resulted in δ = −0.66, whereas differences between the ipsilateral hemispheres at 24 h and 7 days after TBI were characterized by δ = −0.54.

### 2.4. Molecular Dynamics Simulation of PV

To elucidate the molecular mechanisms underlying the interaction between Cx43 and PV, as well as the role of the ionic environment and pH in complex formation, molecular dynamics simulation (MDS) was performed. The simulations were carried out under physiologically relevant conditions with variation in Ca^2+^ ion concentration and pH values, which enabled assessment of the stability of the Cx43–PV complex, the nature of intermolecular interactions, and their energetic dynamics over time. Particular emphasis was placed on the contribution of electrostatic and van der Waals interactions, as well as on evaluating the propensity of the system for self-assembly based on analysis of the energy drift of the complex.

**Figure 5:**
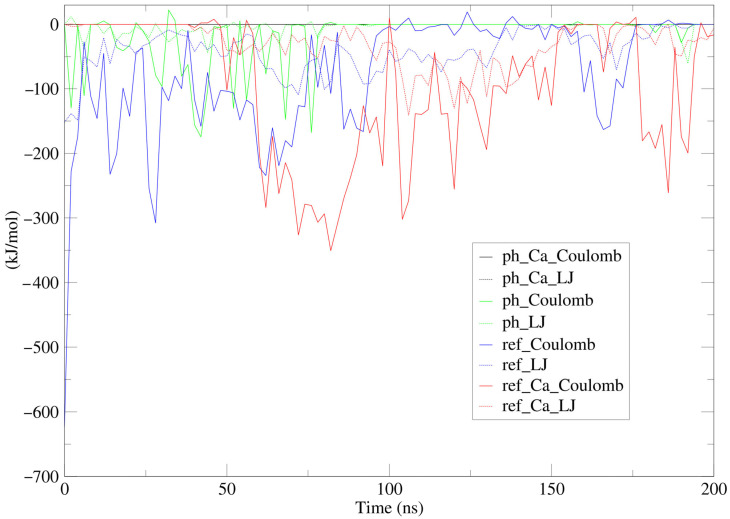
Dynamics of interaction energies between Cx43 and PV during molecular dynamics simulation. Solid lines indicate Coulombic interaction energies, whereas dashed lines represent the contribution of van der Waals interactions (described by the Lennard–Jones potential). The legend labels denote the simulation conditions: ref—reference system at physiological pH; ph—system simulated under reduced (acidic) pH conditions; Ca—system under acidic pH conditions in presence with Ca^2+^ ions; Coulomb—Coulombic interaction energies; LJ—van der Waals interaction energies calculated using the Lennard–Jones potential.

Figure 5 illustrates the changes in interaction energies between Cx43 and PV under different conditions, while the mean values are summarized in Table 1.

**Figure 6:**
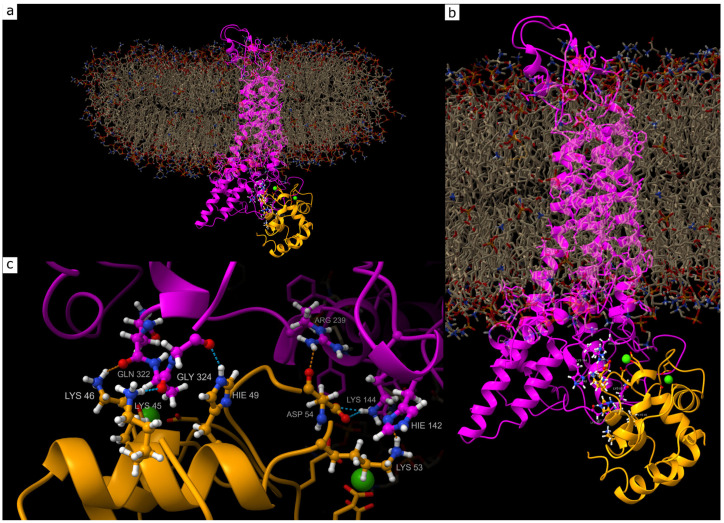
Structure of the most stable PV–Cx43 complex. (**a**)—Full-size visualization of connexin Cx43 (purple) integrated into the membrane in complex with parvalbumin PV (orange). (**b**)—Magnified fragment of the complex. (**c**)—Hydrogen bonds between parvalbumin (orange), coordinating calcium ions (green spheres), and connexin Cx43 (purple). Alphanumeric labels indicate the type and number of amino acid residues in the protein structures. Hydrogen bonds with an overlap value of 0.075 Å are shown in blue, whereas those slightly exceeding the overlap criterion are shown in orange.

Analysis of the mean values and temporal dynamics of interaction energies allows an unambiguous conclusion that the most stable complex is formed between Cx43 and PV in the presence of two Ca^2+^ ions. In the absence of Ca^2+^ ions or under reduced pH conditions, deformation of protein geometries prevents the formation of a stable complex. The conditions of the molecular dynamics simulation made it possible to observe self-organization of the complex between Cx43 and Ca^2+^-bound PV, as reflected by the energy drift values shown in Table 1. The negative energy drift indicates a tendency of the complex toward self-assembly, in contrast to the other modeled systems. At a temperature of 310 K (37 °C), thermal motion of water molecules and ions reduces complex stability, which is evident from the extremely low propensity for self-assembly in the presence of elevated Ca^2+^ concentrations. The structure of the most stable complex is shown in Figure 6.

In the most stable Cx43–PV complex model, formation of an extensive hydrogen-bond network was identified between several amino acid residues localized within the regulatory intracellular domains of Cx43 and the Ca^2+^-binding structural domain of PV. In particular, the LYS46 residue of PV, belonging to the N-terminal region of the Ca^2+^-binding domain, forms hydrogen bonds with GLN322 of Cx43, which is part of the C-terminal cytoplasmic domain of Cx43. It should be noted that these interactions slightly exceed the overlap criteria; however, this is not critical in the context of long-term MDS results. A similar type of hydrogen bonding was observed between ASP54 of PV and ARG239 of Cx43, the latter being localized within the intracellular loop region of the protein. Typical hydrogen bonds with an overlap value of 0.075 Å are formed between LYS45 of PV and GLN322 of Cx43, as well as between HIS49 of PV and GLY324 of Cx43; both Cx43 residues belong to the C-terminal regulatory domain. In addition, LYS53 of PV forms a stable hydrogen bond with HIS142 of Cx43, located in the proximal region of the intracellular loop, with an overlap value of 0.075 Å. The ASP54 residue of PV is also involved in an interaction with LYS144 of Cx43, which is likewise characterized by a typical overlap of 0.075 Å (Figure 6c). The atomic coordinates of the complex can be found in Supplementary Materials: File S1.

The instability of the complex in the presence of a large number of Ca^2+^ ions can be explained by the influence of these ions on the native geometry of PV. It is evident that excessive Ca^2+^ concentrations exert a destabilizing effect on the aspartate- and glutamate-rich calcium-binding domains, which alters the overall geometry of PV and prevents proper spatial arrangement of key amino acid residues required for efficient interaction with Cx43. This hypothesis is supported by the close spatial proximity of the calcium-binding domains of PV to the Cx43–PV interprotein contact region.

## 3. Discussion

The post-traumatic period following TBI is accompanied by a cascade of complex molecular and cellular events that lead to long-lasting disturbances in neuronal and glial homeostasis. One of the key mechanisms of secondary injury is a pathological increase in intracellular Ca^2+^ concentration, which triggers excitotoxicity, oxidative stress, and proteolytic cascades [24]. In response to this calcium imbalance, compensatory mechanisms aimed at maintaining ionic equilibrium are activated. PV, one of the major fast-acting Ca^2+^-buffering proteins in the brain, is predominantly expressed in a population of PV-INs and plays a critical role in the precise temporal coordination of inhibitory networks [8]. After TBI, both the level and functional activity of PV are substantially altered, which may contribute to neuronal death [9,10]. In addition, a potential relationship between PV and Cx43—the principal protein of astrocytic GJs and hemichannels—has recently been proposed [17]. However, the mechanisms underlying this interaction, likely mediated through extensive GJ networks and possibly through secreted or contact-dependent signaling pathways, remain largely unexplored.

In the present study, we performed a comprehensive analysis of the expression and localization of PV and Cx43 in brain tissue after TBI and investigated possible mechanisms of their interaction using in silico approaches. The obtained data revealed a mirror-opposite pattern of these proteins during the acute and subacute phases of the post-traumatic period. In intact nervous tissue, PV and Cx43 expressions were maintained at basal physiological levels. Specifically, PV was predominantly localized in the cytoplasm and neuronal processes, as well as in the nuclei of individual cells, whereas Cx43 appeared as diffusely distributed punctate structures reflecting normal astrocytic GJ function. Additional analysis using the neuronal marker NeuN confirmed that PV-positive cells under physiological conditions belong exclusively to the neuronal population, emphasizing the neuron-specific nature of the observed post-traumatic changes in PV. These findings are consistent with previous studies demonstrating that PV is mainly expressed in cortical interneurons [8].

In the acute phase, 24 h after TBI, a sharp activation of PV expression was observed in the injured area, manifested by a significant increase in fluorescence intensity and an elevated colocalization coefficient, indicating enhanced translocation of PV into neuronal nuclei. Three-dimensional reconstruction of Z-stacks confirmed marked accumulation of PV not only in the cytoplasm and neuronal processes but also within nuclear structures, suggesting its involvement in transcriptional regulation or protection against Ca^2+^-mediated excitotoxicity. Importantly, nuclear localization of PV exhibited a pronounced spatial pattern, being most prominent in cortical regions adjacent to the injury focus and gradually diminishing with increasing distance from the lesion. This observation indicates a local, stress-induced nature of the response. The increase in PV expression likely represents a compensatory intracellular Ca^2+^-buffering mechanism aimed at inhibiting excessive depolarization and preventing apoptotic cascades initiated by glutamate release. Notably, a recent study demonstrated that as early as 3 h after TBI, PV-positive interneurons are selectively activated [25], which may provide an initial limitation of excitotoxicity. This early response may allow surviving neurons, by 24 h post-injury, to initiate their own program of PV expression and nuclear translocation as a secondary protective system against the expanding front of molecular and cellular processes associated with secondary injury. It is well established that PV can effectively reduce cytotoxic Ca^2+^ levels and exert neuroprotective effects [26]. During neurotrauma, including TBI, massive glutamate release leads to excessive activation of NMDA receptors, resulting in Ca^2+^ overload and initiation of apoptotic signaling cascades [27,28,29]. PV plays a key protective role by inhibiting NMDA receptor–induced apoptotic signaling through stabilization of intracellular Ca^2+^ levels, thereby preventing excitotoxicity and promoting neuronal survival [30,31].

In contrast to the increase in PV levels at 24 h post-injury, Cx43 expression during the same phase decreased catastrophically, reaching levels more than fourfold lower than control values, with complete disappearance of characteristic punctate structures. This finding indicates rapid disruption of astrocytic GJs. Such a pronounced dissociation between neuronal and glial responses to traumatic injury suggests stress-specific cellular reactions, in which temporary weakening of astrocytic support enhances the importance of PV-dependent neuronal mechanisms for maintaining Ca^2+^ homeostasis.

The marked depression of Cx43 expression may result from the expanding front of secondary injury processes, including neuroinflammation, oxidative stress, and cell death, as well as from direct mechanisms of Cx43 degradation. Extensive cell death in the injury zone is supported by severe destructive changes, including nuclear fragmentation, pyknotic shrinkage, and reduced nuclear density, reflecting intense loss of neuronal and glial cells during the acute post-traumatic phase. These findings are fully consistent with our recent study, in which we demonstrated that Cx43 expression after TBI exhibits a pronounced transient pattern: an initial critical decline at 24 h post-injury followed by a reversal toward aggressive hyperexpression with accumulation of Cx43-associated clusters and formation of protein aggregates under conditions of reactive astrogliosis [22].

The near-complete loss of Cx43 expression reflects critical disruption of GJ network integrity, thereby promoting progressive disturbances in neuroglial homeostasis. The reciprocal, differentiated response of Cx43 and PV highlights complex complementary mechanisms between these two systems, wherein temporary weakening of glial support enhances the role of PV-dependent neuronal mechanisms in maintaining Ca^2+^ homeostasis. Cx43 is known to play a central role in ionic homeostasis, redistribution of energy substrates during heightened neuronal activity, and regulation of synaptic transmission [32]. Astrocytic Cx43 reduces pathological extracellular Ca^2+^ levels [33], thereby protecting neurons from Ca^2+^-mediated excitotoxicity, and regulates the propagation of calcium waves [34]. In models of stroke, reduced Cx43 expression under conditions of increasing Ca^2+^ concentration has been shown to negatively correlate with neuronal survival [35].

Transition to the subacute phase, assessed at 7 days after TBI, revealed a pronounced inversion in the expression pattern of PV and Cx43. PV levels in the injury zone sharply declined both relative to control and compared with the 24 h post-injury time point. Moreover, nuclear localization of PV almost completely disappeared, along with cytoplasmic localization, which was only sporadically detected in individual neurons. Against the background of loss of intracellular PV immunoreactivity at later time points after TBI, accumulation of PV-immunoreactive material in the extracellular space was observed. Three-dimensional analysis revealed a fine-granular dispersion, while two-dimensional immunofluorescence microscopy showed a persistent increase in diffuse background signal within the neuropil. This phenomenon, most pronounced at 7 days post-injury, likely reflects the release of PV from damaged or dying neurons and its accumulation in the extracellular milieu during progressive secondary injury.

The dramatic decrease in PV levels at 7 days after TBI is likely caused by depletion or selective loss of PV-positive interneurons. These findings are consistent with previous studies reporting a pronounced reduction in PV immunoreactivity at later stages after TBI [36,37]. In a mouse model of controlled cortical impact, a marked decrease in PV expression was observed as early as 14 days post-injury, with the loss of PV immunoreactivity exceeding the actual death of genetically labeled PV interneurons. This suggests preferential degradation or suppression of PV synthesis rather than cell loss alone [36]. A similar negative trend toward reduced expression and loss of PV-positive interneurons persisted at later time points, as clearly demonstrated in the thalamus 6 months after TBI, indicating a systemic and long-lasting impairment of GABAergic inhibition [37]. Moreover, postmortem analyses of human brain tissue from individuals who died after TBI have shown that a reduction in PV-positive neurons is associated with markers of oxidative stress [38]. Notably, sustained oxidative stress is a major component of secondary brain injury after TBI [39]. Clinical studies in TBI patients have demonstrated that markers of oxidative damage remain elevated not only during the acute but also during the subacute post-traumatic phase, persisting for up to one week after injury [40]. Prolonged oxidative stress may therefore contribute to the loss of PV-positive neurons under conditions of traumatic stress.

In contrast, Cx43 expression during this period increased markedly, exceeding control values by 2.2-fold and the acute-phase Cx43 level by nearly tenfold. This increase was accompanied by pathological qualitative changes manifested as the formation of large aggregates and elongated linear accumulations. Such hyperexpression and aggregation of Cx43 likely reflect the development of reactive gliosis, in which proliferating astrocytes enhance intercellular communication to facilitate clearance of cellular debris, restoration of the blood–brain barrier, and support of surviving neurons. Although destructive nuclear changes persisted, nuclear density within the injury zone began to increase, which can be interpreted as a consequence of inflammatory cell infiltration and proliferation of reactive glia, signaling the initiation of reparative processes.

For a visual representation of the identified mirror-opposite patterns of PV and Cx43, including their changes in various phases, key observations, and connections to modern research, we have summarized the data in Table 2. This allows for a quick comparison of phase shifts and emphasizes the transition from neuronal protection to glial remodeling, which is consistent with the general mechanisms of response to TBI.

The observed mirror-opposite pattern of PV and Cx43 reveal a complex molecular and cellular response to TBI, in which the acute phase is characterized by stabilization of neuronal ionic homeostasis via PV, whereas the subacute phase is dominated by glial remodeling mediated by Cx43. These oppositely directed transient changes illustrate a transition from Ca^2+^-mediated excitotoxicity and cell death toward recovery and regeneration. The present findings are consistent with previous studies demonstrating the neuroprotective role of PV and the involvement of Cx43 in gliosis, while at the same time uncovering more complex mechanisms of their temporal changes and spatial reorganization. Notably, pharmacological suppression of PV^+^ interneuron activity at the time of injury has been shown to significantly increase neuronal survival and reduce astrogliosis at 7 days after TBI [25], suggesting the existence of subtle feedback regulatory mechanisms between PV and Cx43 as the principal protein of astrocytic GJs.

In addition, we observed a reduction in the number of NeuN^+^ nuclei within the injured zone at 24 h after TBI, reflecting a substantial loss of the neuronal phenotype during the acute phase of the post-traumatic period. This decrease was accompanied by characteristic morphological features of apoptosis, indicating extensive cell death. The spatiotemporal coincidence of the peak reduction in NeuN immunoreactivity with nuclear translocation of PV and a sharp decrease in Cx43 expression indicates profound dysfunction of neuroglial interactions during the acute phase of TBI. Partial restoration of the proportion of NeuN^+^ cells at 7 days after injury, accompanied by a reduction in nuclear destructive changes, suggests a transient loss of NeuN immunoreactivity in a subset of neurons. At the same time, incomplete recovery of the NeuN^+^ population confirms irreversible loss of a fraction of neurons and the establishment of persistent post-traumatic cortical alterations. These results are consistent with studies demonstrating that loss of NeuN immunoreactivity after TBI does not always reflect direct neuronal death, but may represent a transient downregulation of NeuN expression in functionally impaired yet still viable neurons at early time points, with subsequent recovery of the neuronal phenotype [41]. It has also been shown that disruption of neuronal membranes after TBI is biphasic and may persist into subacute and chronic periods, during which a subset of neurons with delayed membrane damage lose NeuN immunoreactivity without overt signs of cell death [42].

An important complement to the described molecular and cellular changes was the quantitative assessment of the spatial organization and density of Cx43-associated cluster structures using automated image analysis methods. Given the pronounced heterogeneity of fluorescent signals characteristic of injured tissue, as well as the transient nature of Cx43 expression, traditional manual annotation proved to be limited in reproducibility and sensitivity, particularly under conditions of low-intensity or partially saturated signals. Accordingly, the present study employed a detector based on the DINO-SwinL architecture, enabling standardized quantification of Cx43-associated clusters and reducing the influence of subjective annotation factors.

The results demonstrated that variability in imaging conditions and partial signal saturation indeed constrained annotation consistency, thereby defining a practical upper bound for detection accuracy. Such uncertainty is widely described for fluorescence microscopy data and likely underlies a portion of the observed detection errors [43,44]. Nevertheless, the achieved mAP value (78.4%) reflects a balance between model capacity and biologically driven annotation noise characteristic of post-traumatic specimens. Notably, even with a relatively limited training dataset, the model exhibited stable and reproducible performance, in some cases identifying Cx43 clusters more consistently than a human operator, particularly in regions with low-intensity signals.

From a biological perspective, this observation is of particular importance. The model appears to rely not only on absolute intensity thresholds but also on morphological and contextual features characteristic of Cx43-associated structures, allowing partial averaging of human annotation noise and recovery of stable visual patterns. A similar effect has been described in noise-robust learning and suggests that automated analysis methods may, in certain cases, more reliably reflect the true organization of biological structures than individual manual annotation [45].

Statistical analysis of the number of Cx43-associated clusters revealed clear differences between experimental groups, with the ipsilateral injury zone at 7 days after TBI consistently exhibiting a higher number of aggregated Cx43-associated clusters compared with other conditions. These differences remained significant after correction for multiple comparisons, indicating a specific pattern of astrocytic network remodeling during the subacute phase of the post-traumatic period. The combination of a reduced number of functionally organized Cx43 structures with their morphological aggregation suggests that, at this stage, processes of structural reorganization and reactive gliosis predominate over mechanisms of ionic homeostasis.

Integration of immunohistochemical analysis with machine vision approaches not only quantitatively confirmed the transient and phase-dependent changes in Cx43 but also expanded understanding of its spatial organization after TBI. Together with the identified mirror-opposite pattern of PV, these data underscore the existence of a multilevel, temporally coordinated neuron–glia adaptation, in which early PV-dependent stabilization of Ca^2+^ homeostasis is followed by Cx43-mediated glial remodeling. In the future, incorporation of longitudinal analyses with additional time points, as well as application of deep learning–based segmentation models, may enable more precise quantitative characterization of Cx43 cluster recovery and further elucidate their functional role in TBI outcomes.

It is now well established that Cx43 can be expressed not only in astrocytes but also in neurons. Notably, approximately 40% of PV^+^ neurons have been reported to express Cx43 [15]. GJs are known to mediate communication between different GABAergic neurons and serve as a critical element in maintaining their normal metabolism and synchronization of neuronal activity [19]. To investigate the direct molecular mechanisms underlying the interaction between PV and Cx43, we performed molecular dynamics simulations (MDS) to evaluate interaction energies under various conditions, including the presence of Ca^2+^ ions and changes in pH. These conditions are particularly relevant for post-traumatic states, in which disturbances of Ca^2+^ homeostasis and acidosis play a central role. Metabolic alterations, including hypoxia, lactate accumulation, and reduced tissue buffering capacity, lead to acidification of the injured area after TBI [46], with pH values reported to decrease to critically low levels of approximately 6.5 [47]. Such pH-associated shifts are known to induce significant conformational changes in the architecture of many proteins, including Cx43 [48] and PV [49], thereby directly affecting their functional properties.

Analysis of these data allows an unambiguous conclusion that the most stable complex is formed in the model containing two Ca^2+^ ions. Under these conditions, the lowest Coulombic interaction energy is observed compared with other models, without compromising the stabilizing contribution of van der Waals interactions (described within the Lennard–Jones potential formalism). A key indicator of stability is the negative energy drift, which reflects a tendency of the complex toward self-organization under the influence of Ca^2+^. This is consistent with the known ability of Ca^2+^ to bind PV and induce conformational changes that promote its association with Cx43.

At the structural level, this stability is ensured by the formation of specific interactions between the Ca^2+^-binding domain of PV and the regulatory intracellular domains of Cx43. In the most stable model, a network of hydrogen bonds was identified between N-terminal residues of PV (LYS45, LYS46, HIS49, LYS53, and ASP54) and residues of the C-terminal cytoplasmic domain and intracellular loop of Cx43 (GLN322, GLY324, HIS142, LYS144, and ARG239). The presence of both typical hydrogen bonds with an overlap value of 0.075 Å and bonds slightly exceeding the overlap criterion indicates a dynamic molecular complex that can rapidly adapt to Ca^2+^-induced conformational changes in PV. It is noteworthy that Ca^2+^ binding to PV is characterized by extremely high affinity, with association constants reaching up to 10^10^ M^−1^, ensuring rapid and efficient Ca^2+^ sequestration under physiological conditions. This binding is accompanied by pronounced conformational rearrangements in the PV structure, particularly in β-isoforms, including helix rotation, reorganization of the hydrophobic core, and alterations in interdomain contacts, which dramatically increase the thermodynamic stability of the protein [50]. Such Ca^2+^-dependent conformational changes contribute to PV stabilization against oxidative stress and determine its high Ca^2+^ affinity as a prerequisite for maintaining its active conformation [51].

In contrast, in the Ca^2+^-free model, interaction energies are less favorable and exhibit a positive energy drift, indicating instability and a tendency toward dissociation. Under reduced pH conditions, interaction energies are markedly weakened and also display a positive drift, suggesting deformation of protein geometries that prevents complex formation, likely due to protonation of key residues and disruption of ionic bridges. It has been reported that decreased pH activates acid-activated proteases, inducing PV proteolysis and reducing its immunological properties. Moreover, low pH causes structural alterations in PV, including a reduction in α-helical content, an increase in β-turns and random coils, and decreased surface hydrophobicity, leading to disruption of conformational epitopes and reduced IgE-binding capacity [49]. Considering the identified hydrogen-bond network, it can be hypothesized that protonation of acidic and histidine residues in PV and Cx43 directly destabilizes interdomain interactions that are critical for maintaining the complex.

The most dramatic absence of interaction was observed in the model combining reduced pH with Ca^2+^, where interaction energies were essentially zero and no energy drift was detected. This finding confirms that the combination of acidosis and Ca^2+^ completely suppresses association, possibly due to competing effects on PV conformation. Simulation conditions at 37 °C mimic the physiological environment, in which thermal motion of water molecules and ions reduces complex stability, particularly in the presence of elevated Ca^2+^ concentrations. This observation is consistent with the Ca^2+^ overload characteristic of the acute phase of TBI, which may disrupt PV–Cx43 interactions and contribute to suppression of Cx43 function.

These molecular dynamics data complement the immunofluorescence observations by providing a mechanistic explanation for why PV activation as a Ca^2+^ buffer during the acute phase of TBI correlates with suppression of Cx43. Transient Ca^2+^ elevation may initially stabilize the interaction, but subsequent acidosis and Ca^2+^ overload lead to its destabilization, thereby exacerbating glial dysfunction. The preferential localization of interactions within the regulatory intracellular domains of Cx43 further suggests a potential influence of PV on signaling and phosphorylation sites of Cx43, which may additionally modulate its functional state.

During the subacute phase, hyperexpression of Cx43 may reflect a compensatory response mediated through alternative pathways independent of PV. Overall, integration of these findings highlights the central role of Ca^2+^ and pH in regulating the PV–Cx43 complex as a key factor in TBI pathogenesis, and suggests new potential therapeutic targets, such as stabilizers of calcium homeostasis or pH modulators, for preventing secondary brain injury.

## 4. Materials and Methods

### 4.1. Animals and Ethical Approval

Adult male CD-1 mice aged 14–15 weeks and weighing 20–25 g were used in the study. Animals were housed in groups of 6–7 per cage with ad libitum access to food and water. Environmental conditions were maintained at a stable level, with room temperature of 22–25 °C and a ventilation system providing approximately 18 complete air exchanges per hour.

All experimental procedures were performed in accordance with international and national guidelines for the humane treatment of laboratory animals. The study complied with EU Directive 86/609/EEC (24 November 1986) as well as Russian regulatory documents, including the “Rules of Laboratory Practice” (Order of the Ministry of Health of the Russian Federation No. 708n, 23 August 2010) and GOST 33215–2014 governing animal housing and experimental procedures. Protocol No. 2 was reviewed and approved by the Bioethics Committee of Don State Technical University on 17 February 2020.

### 4.2. Object and Procedure

To reproduce severe focal TBI, a modified penetrating focal cortical injury model was used, based on a directed weight drop with controlled penetration of the tip into brain tissue and belonging to the group of focal injury models analogous to controlled cortical impact [52,53]

Anesthesia was induced by intramuscular injection of a mixture of Xyla (0.2 mL/kg, 2% xylazine hydrochloride solution; Interchemie Werken “de Adelaar” BV, Venray, The Netherlands) and Zoletil (15 mg/kg, tiletamine–zolazepam combination; Virbac, Carros, France). Adequate anesthesia depth was verified by the absence of response to painful stimuli and suppression of the corneal reflex.

Prior to surgery, scalp hair was removed and the skin was disinfected. The animal was fixed in a stereotaxic frame ensuring reproducible head positioning. A midline incision was performed to expose the skull, and a circular craniotomy (3 mm diameter) was created in the parietal region using a dental drill.

Through the opening, impact was delivered by a 150 g metal rod with a 2 mm diameter and 3 mm length tip moving inside a vertical guiding system that eliminated manual variability. The rod was released from a fixed height of 1 cm, and the tip penetrated the dura mater and cortex, producing a reproducible focal injury. Standardization of mass, drop height, tip geometry, and head fixation ensured consistent injury severity in accordance with controlled weight-drop and controlled cortical impact principles [54].

The impact site was defined using stereotaxic coordinates (2 mm posterior to bregma and 1 mm lateral to the midline), corresponding to the parietal cortex. For subsequent analyses, tissue samples were obtained from the ipsilateral perilesional parietal cortex surrounding the injury site while excluding the necrotic core. Regions of interest were selected at a consistent distance from the lesion in all animals to ensure anatomical comparability between groups. After injury induction, the wound was rinsed with saline, the opening was sealed with bone wax, and the skin was sutured.

**Figure 7:**
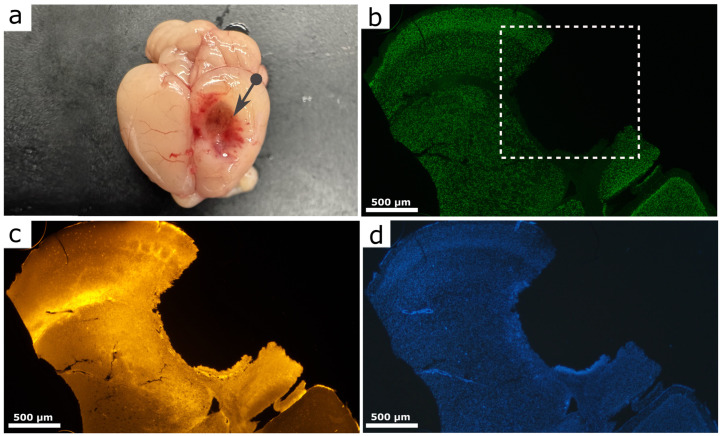
Localization of the injury site and histological characterization of the lesion area. (**a**)—Mouse brain after traumatic brain injury; the gray arrow indicates the impact site. (**b**)—Histological section stained for NeuN the white dashed rectangle outlines the lesion area visible in tissue sections. (**c**)—The same section stained for PV showing the corresponding region of interest. (**d**)—Nuclear staining with Hoechst highlighting overall cellular distribution within the lesion area. Scale bar: 500 μm.

The localization and morphological characteristics of the lesion area were verified histologically using staining with the neuronal marker NeuN, PV, and nuclear labeling with Hoechst. These markers allowed identification of the impact site, delineation of the necrotic core boundaries, and visualization of the surrounding perilesional cortex used for subsequent analyses. Representative images illustrating the macroscopic injury location and the corresponding histological region of interest are shown in Figure 7.

### 4.3. Confocal and Optical Fluorescence Microscopy

The localization of PV and Cx43 proteins in the mouse brain was assessed at 24 h and 7 days after TBI using the following protocol. Animals were deeply anesthetized and transcardially perfused via the right ventricle with 4% paraformaldehyde (PFA). The perfusion needle was inserted into the right ventricle, and the right atrium was incised to allow outflow. Under these conditions, the fixative passes through the pulmonary circulation, enters the left heart and aorta, and subsequently distributes throughout the systemic vasculature including cerebral vessels, which corresponds to vascular transcardial perfusion fixation widely used in rodents [55,56]. This procedure ensured uniform fixation of brain structures.

To enhance PFA penetration and minimize artifacts, animals were kept in an inverted head-down position for 2 h, facilitating gravitational flow of the fixative. After brain extraction, tissues were additionally fixed in fresh 4% PFA for 12 h to equalize fixation gradients and ensure complete crosslinking of proteins following vascular perfusion.

Frontal brain sections approximately 0.4 cm thick, specifically including areas of necrotic damage induced by TBI, were prepared. Thin sections (~20 μm) were obtained using a high-precision vibratome (Leica VT 1000 S, Leica Biosystems, Nussloch, Germany) and subsequently cryoprotected by sequential incubation in 15% and 30% sucrose solutions for 1 h each. Sections were then frozen at −80 °C for long-term storage.

Tissue sections were extensively washed in phosphate-buffered saline (PBS) to completely remove residual fixative and minimize nonspecific background staining. They were then incubated for 1 h at room temperature in a blocking solution containing 5% bovine serum albumin (BSA, Sisco Research Laboratories Pvt. Ltd., Mumbai, India) and 0.3% Triton X-100 (Sisco Research Laboratories Pvt. Ltd., Mumbai, India). This step effectively reduced nonspecific antibody binding, thereby improving the specificity and reliability of the subsequent immunohistochemical labeling.

To investigate colocalization of PV and Cx43, sections were incubated for 48 h at 4 °C with a mixture of primary antibodies: mouse anti-PV (1:100, P3088; Sigma-Aldrich, St. Louis, MO, USA) and rabbit anti-Cx43 (1:100; E-AB-70097; Elabscience Biotechnology Inc., Houston, TX, USA). In additional experiments, after initial exposure to anti-PV antibodies, sections were co-incubated with rabbit anti-NeuN antibodies (1:1000) to determine the neuronal localization of PV.

Following multiple rigorous washing steps in PBS to eliminate unbound primary antibodies, sections were incubated with secondary fluorescent conjugates. For confocal microscopy, the following were used: anti-rabbit IgG (H+L) Abberior STAR 635P (1:500, Abberior GmbH, Göttingen, Germany) or anti-rabbit Alexa Fluor 488 (1:500; ab150077, Abcam, Cambridge, UK), together with anti-mouse IgG (H+L) Abberior STAR 580 (1:500, Abberior GmbH, Göttingen, Germany) or anti-mouse Alexa Fluor^®^ 647 (1:500; ab150115, Abcam, Cambridge, UK). For conventional fluorescence microscopy, rabbit antibodies conjugated to Alexa Fluor 488 (1:500; ab150077, Abcam, Cambridge, UK) and mouse antibodies conjugated to Alexa Fluor 555 (1:500; ab150114, Abcam, Cambridge, UK) were applied.

Negative controls consisted of sections processed identically but without primary antibodies. Nuclear counterstaining of neurons and glial cells was performed using Sytox Green Stain (ThermoFisher Scientific, Waltham, MA, USA) diluted 1:1000 in PBS for confocal imaging, or Hoechst 33342 for fluorescence microscopy. Sections were stained for 20–30 min at room temperature in complete darkness to prevent photobleaching and preserve signal intensity. Following staining, sections underwent three additional washes in PBS. Prepared slides were mounted in anti-fade mounting medium (Abberior GmbH, Göttingen, Germany) and coverslipped.

Imaging was performed using a state-of-the-art inverted confocal laser scanning microscope, Abberior Facility Line (Abberior Instruments GmbH, Germany), which provided ultra-high-resolution visualization of intra- and intercellular structures suitable for subsequent 3D reconstruction. Three-dimensional models were generated via Z-stack acquisition with a step size of 200 nm and a pixel size of 40 nm. Image processing and 3D reconstruction were carried out using ImageJ software (version 1.54j, National Institutes of Health, Bethesda, MD, USA). Fluorescence microscopy was conducted on an Olympus BX53 microscope (Olympus Corporation, Tokyo, Japan) equipped with a high-resolution digital camera (EXCCD01400KPA, Hangzhou ToupTek Photonics Co., Ltd., Hangzhou, China).

Quantitative analysis of PV expression levels in fluorescence microscopy images, where red (Cx43) and yellow (PV) signals were visualized, was performed using ImageJ (version 1.54j). The entire field of view was selected as a rectangular region of interest, and mean fluorescence intensity of the target signal was calculated. Background intensity was measured in regions lacking specific signal and subtracted from the mean signal intensity, followed by normalization using the formula:

$$\mathrm{Relative}\mathrm{Fluorescence}\mathrm{Intensity}(\%)=\left(\frac{{Mean}_{signal}{-Mean}_{background}}{{Mean}_{background}}\right)\times 100$$

Values were expressed as percentages relative to background intensity.

For assessment of the neuronal phenotype, quantitative analysis of NeuN-positive nuclei was performed. The proportion of NeuN^+^ nuclei was calculated as a percentage of NeuN-positive nuclei relative to the total number of Hoechst-positive nuclei using the formula:

$$\mathrm{NeuN}\mathrm{index}(\%)=\left(\frac{{NeuN}^{+}}{{Hoechst}^{+}}\right)\times 100$$

Colocalization of NeuN and Hoechst signals was additionally assessed using ImageJ (version 1.51r; http://rsb.info.nih.gov/ij/, accessed on 10 February 2017) with the JACoP plugin, where the M1 coefficient was calculated to provide a quantitative metric of spatial overlap between signals.

### 4.4. Dataset Collection and Annotation Protocol

**Figure 8:**
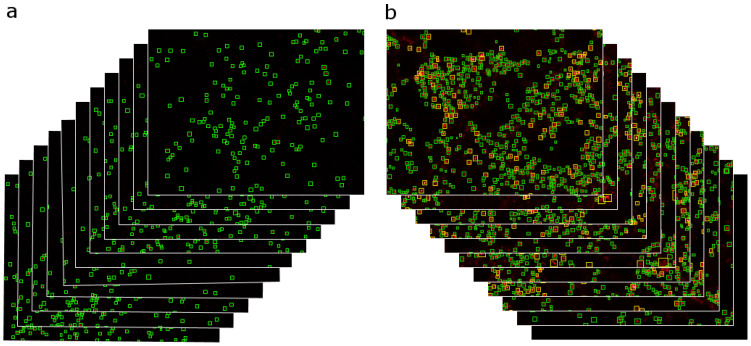
Representative confocal Z-stacks obtained from the contralateral (**a**) and ipsilateral (**b**) hemispheres 7 days after TBI. Regular Cx43 puncta are outlined in green, and aggregated puncta in yellow.

Images were acquired from the contralateral and ipsilateral hemispheres under sham-operated conditions and at 1 and 7 days after TBI. The complete dataset comprised 38 three-dimensional Z-stacks containing a total of 1699 two-dimensional frames. Examples are shown in Figure 8.

Manual annotation was performed on 206 images using the makesense.ai platform (https://www.makesense.ai/, accessed on 15 March 2024) by a single operator. Half of these images were used for model training, while the remaining half were reserved exclusively for evaluation. To prevent data leakage, no individual Z-stack was split between the training and test sets. Frames were randomly sampled across the full depth of each Z-stack to ensure exposure of the model to the complete variability of subcellular organization.

The main challenge during annotation was heterogeneity in image brightness. Some Z-stacks contained overexposed regions in which Cx43 signals appeared saturated, whereas others exhibited adequate signal intensity. These variations, together with the inherent uncertainty of manual human annotation, influenced decisions on whether structures should be labeled as “aggregated” Cx43. Saturated, abnormally bright clusters or tightly packed punctate structures that appeared to merge into larger domains were also assigned to the aggregated class. Annotation criteria for Cx43 puncta were based on assessments of physical size, expected morphology, and fluorescence intensity. Specifically, normal punctate structures were defined as approximately 0.35–0.50 μm (200–300 nm at 40 nm/pixel), whereas structures larger than 300 nm or exhibiting saturated “white” fluorescence were classified as aggregated.

### 4.5. Model Training

Model training was performed using the best_quality preset of the AutoGluon framework (autogluon.multimodal 1.1.1) implemented in the Python programming language (version 3.10.12) [57]. This preset is designed to achieve maximal predictive performance and incorporates a predefined set of state-of-the-art algorithms and training strategies for computer vision tasks.

The preset is based on the DINO-SwinL architecture [58,59], which combines the DINO object detector with a Swin-Large transformer backbone. This architecture provides high representational capacity and efficient extraction of informative features, which is particularly important for complex object detection tasks requiring high accuracy and robustness to data variability.

Model training was conducted for 100 epochs with a batch size of 2, enabling stable parameter optimization under computational resource constraints. Standard data augmentation techniques, including random image flipping and rotation, were applied during training to enhance model generalization and reduce the risk of overfitting.

Hyperparameter tuning was performed automatically using AutoGluon’s internal optimization pipeline, which includes systematic parameter search and evaluation without the need for manual intervention. This approach minimized the influence of subjective factors during model configuration and ensured reproducibility of the obtained results.

### 4.6. Molecular Dynamics Simulation

For molecular dynamics simulation (MDS), the geometries of human PV [60] and a single subunit of Cx43 [61] were retrieved from the UniProt database. The Cx43 model was completed to its full-length structure using the boltz-2 program [62] and embedded into a model lipid bilayer membrane using the packmol-memgen utility [63]. In the first step, docking between apo-PV (without Ca^2+^ ions in the corresponding domains) and Cx43 was performed using the haddock3 program [64]. A total of three docking models were obtained, of which only one was considered biologically relevant (in the remaining complexes, PV bound to the transmembrane domain).

The geometry of this complex was used as the reference (ref) structure for molecular dynamics simulation over 200 ns at a temperature of 310 K (37 °C), with K^+^ and Cl^−^ ion concentrations sufficient to neutralize the system but not less than 140 mmol/L. Because the complex dissociated during the 200 ns simulation, three additional geometries were constructed based on the coordinates of the centers of mass of the proteins in the dissociated complex. Thus, three models were generated:

1. parvalbumin containing two Ca^2+^ ions in the corresponding binding domains (ref_Ca);
2. parvalbumin and connexin under reduced pH conditions characteristic of ischemia (pH = 6.3), corresponding to protonation of histidine residues (ph);
3. the ph model with an additional 10 Ca^2+^ ions, corresponding to Ca^2+^ influx into the neuronal cytoplasm during ischemia (ph_Ca).

In all cases, the MDS parameters were as follows: AMBER force fields were used (ff19SB for proteins [65], lipid21 for lipids [66], and OPC for water and ions [67]). Simulations were performed at 310 K and 1 atm pressure. All geometries underwent energy minimization and multistep equilibration in temperature and pressure prior to 200 ns production MDS. Based on the resulting trajectories, mean interaction energies between PV and the Cx43 hemichannel were calculated, and the dynamics of hydrogen bond formation between these proteins were analyzed.

### 4.7. Statistical Analysis

Statistical analysis was performed using one-way analysis of variance (ANOVA) followed by Tukey’s post hoc test. Normality of distribution was assessed with the Shapiro–Wilk test, and homogeneity of variances was evaluated using the Brown–Forsythe test. When the assumptions of normality or homogeneity of variances were violated, the non-parametric Kruskal–Wallis test was applied instead. All data were analyzed in a blinded manner. Differences were considered statistically significant at *p* < 0.05 (n = 6). Results are presented as mean ± standard error of the mean (M ± SEM). Data processing was carried out using SigmaPlot 12.5 (Systat Software Inc., San Jose, CA, USA) and JASP 0.19.1 (University of Amsterdam, The Netherlands).

In addition, all computations and extended analyses related to Cx43 aggregation were performed in the Python environment using the SciPy, NumPy, pandas, and statsmodels libraries. Only groups with available observations were included in the analysis. Because normality was violated, pairwise group comparisons were conducted using nonparametric tests—the Kruskal–Wallis test and the Mann–Whitney U test. Correction for multiple comparisons was applied using the Benjamini–Hochberg false discovery rate (FDR) procedure. A *p* value < 0.05 was considered statistically significant. Effect sizes for pairwise comparisons were calculated as Cliff’s delta (δ) and interpreted according to established thresholds: |δ| < 0.147, negligible; 0.147 < |δ| < 0.33, small; 0.33 < |δ| < 0.474, medium; |δ| > 0.474, large.

## 5. Limitations of the Study

The present study has several limitations that should be considered when interpreting the obtained results.

First, quantitative conclusions regarding protein expression were primarily based on immunofluorescence and immunohistochemical analyses. While these approaches reliably assess spatial localization and relative changes in signal intensity, they do not provide direct biochemical quantification of protein concentration. Therefore, the observed alterations in PV and Cx43 should be interpreted as relative changes in immunoreactivity rather than absolute protein abundance. Independent biochemical methods such as Western blotting or ELISA would further strengthen conclusions regarding total protein levels.

Second, semi-quantitative image analysis depends on imaging conditions and staining quality. In this study identical acquisition parameters were maintained within experimental series and background normalization was applied; however, variability inherent to fluorescence microscopy may influence measured intensity values. Accordingly, fluorescence measurements were used to identify robust phase-dependent trends rather than precise quantitative differences. Three-dimensional reconstructions were employed primarily to confirm spatial redistribution of signals and did not serve as the primary source of quantitative measurements.

Third, molecular dynamics simulations represent a theoretical model of potential PV–Cx43 interaction rather than direct experimental evidence of a stable molecular complex in vivo. The simulations were designed to evaluate physicochemical feasibility of interaction under post-traumatic ionic and pH conditions and therefore should be interpreted as a mechanistic hypothesis explaining the observed inverse expression dynamics. The study does not claim the existence of a permanently assembled complex in specific cell types, and experimental validation of direct binding will be required in future investigations.

In addition, the temporal resolution of the study was limited to two post-traumatic time points (24 h and 7 days). Although these intervals capture acute and subacute phases of injury, intermediate and later stages of protein redistribution may remain undetected. Therefore, the described dynamics should be interpreted as phase-representative rather than continuous temporal trajectories, and future studies including multiple intermediate time points are required to fully reconstruct the progression of PV and Cx43 reorganization.

Finally, the experiments were performed using a controlled focal cortical injury model. While this approach provides high reproducibility of lesion severity, different TBI paradigms may involve distinct neuroglial responses. Consequently, extrapolation of the observed PV–Cx43 dynamics to other forms of traumatic brain injury should be made with caution and requires validation in additional experimental models.

## 6. Conclusions

The present study demonstrates that TBI induces a phase-dependent reorganization of neuronal and astrocytic homeostatic mechanisms characterized by reciprocal dynamics of PV and Cx43. The acute phase is associated with increased neuronal PV expression and nuclear redistribution accompanied by disruption of physiologically organized astrocytic Cx43 structures, whereas the subacute phase is characterized by reduced neuronal PV signal and pronounced aggregation and remodeling of Cx43-positive GJs. These findings suggest a transition from predominantly neuronal calcium-buffer–mediated compensation toward astrocyte-driven network reorganization during post-traumatic adaptation.

Automated analysis of individual optical sections revealed early micro-clustering of Cx43 that precedes the formation of large aggregates detectable by volumetric imaging, indicating a staged process of GJ disassembly followed by structural remodeling. This observation reconciles the apparent reduction in organized Cx43 with the simultaneous emergence of aggregated structures during the acute phase.

Molecular dynamics simulations further indicate that Ca^2+^ concentration and pH critically influence the potential interaction landscape between PV and intracellular domains of Cx43. These calculations were not intended to reconstruct a proven structural complex but to evaluate the physicochemical feasibility of transient interactions under post-traumatic conditions such as calcium overload and acidosis. The modeling therefore provides a mechanistic framework consistent with the observed inverse dynamics of the proteins, rather than direct evidence of stable binding in vivo. Considering the close spatial proximity of PV-positive interneurons and Cx43-containing astrocytic GJ networks, such transient interactions may occur within neuron–astrocyte microdomains or within interneurons themselves.

Together, the results support a model in which neuronal calcium buffering and astrocytic coupling represent temporally coordinated components of the neuroglial response to injury. Understanding this shift from neuronal to glial homeostatic regulation may help explain delayed network dysfunction after TBI and highlights Ca^2+^- and pH-dependent neuroglial signaling as a potential target for future therapeutic modulation.

## Figures and Tables

**Table 1:** Mean values of Coulombic and van der Waals interaction energies (in the Lennard–Jones formalism) in the corresponding models. Mean energy values are presented with standard error, along with the overall energy drift of the Cx43–PV complex during the simulation.

Model	Type of Interaction	Energy ± Err.Est (kJ/mol)	Tot-Drift (kJ/mol)
ref	Coulomb	−76.2043 ± 28.0	188.1020
	Lenard-Jonson	−41.0566 ± 8.8	52.2935
ref_Ca	Coulomb	−96.3931 ± 32.0	−49.0147
	Lenard-Jonson	−30.9422 ± 11.0	−30.5392
ph	Coulomb	−18.5754 ± 11.0	58.8536
	Lenard-Jonson	−4.6380 ± 2	8.5333
ph_Ca	Coulomb	0 ± 0	0
	Lenard-Jonson	0 ± 0	0

**Table 2:** Mirror-opposite patterns of PV and Cx43 expression in various phases of the post-traumatic period after TBI, including key observations, molecular interactions, and connections to recent research.

Phase	PV (Parvalbumin) Changes	Cx43 (Connexin 43) Changes	Key Observations and Interpretation	Association with Recent Studies
Intact (Control)	- Baseline physiological expression level. - Predominantly cytoplasmic localization with distribution in neuronal processes; nuclear localization observed in a subset of cells. - Strictly neuron-specific expression (PV-INs), confirmed by colocalization with NeuN.	- Baseline physiological expression level. - Diffusely distributed punctate structures corresponding to functionally organized astrocytic GJs.	- Maintenance of neuronal–glial homeostasis. - PV provides high-affinity Ca^2+^ buffering and precise temporal coordination of GABAergic networks. - Cx43 sustains inter-astrocytic communication and metabolic/ionic integration.	Basal PV expression in cortical interneurons has been previously described [8]. The role of Cx43 in maintaining ionic homeostasis and intercellular exchange has been confirmed in experimental models [32,33,34].
Acute (24 h after TBI)	- Marked increase in expression and fluorescence intensity. - Pronounced nuclear translocation (most prominent near the lesion core). - Increased colocalization coefficient. - 3D reconstruction confirms accumulation in cytoplasm, processes, and nuclei.	- More than fourfold reduction relative to control. - Disappearance of characteristic punctate structures (disorganization of astrocytic GJs).	- PV upregulation is interpreted as a compensatory Ca^2+^-buffering response under excitotoxic and NMDA-dependent Ca^2+^ overload conditions. - Nuclear localization may reflect involvement in transcriptional regulation or neuroprotection. - Cx43 downregulation indicates disruption of the astrocytic network associated with neuroinflammation and oxidative stress. - Extensive cell death observed (pyknosis, nuclear fragmentation, reduction in NeuN^+^ cells).	Early activation of PV-positive interneurons after TBI has been reported [25]. The neuroprotective role of PV during Ca^2+^ overload has been confirmed experimentally [26,30,31]. Transient Cx43 downregulation and its involvement in secondary injury propagation have been demonstrated in TBI [22] and stroke models [35].
Subacute (7 days after TBI)	- Marked reduction in expression (below control and acute phase levels). - Near-complete loss of nuclear and cytoplasmic localization. - Accumulation of extracellular immunoreactive material (fine granular dispersion and diffuse neuropil signal).	- Significant upregulation (≈2.2-fold above control and ≈10-fold above acute phase). - Formation of large aggregates and elongated linear clusters. - Increased number of Cx43-associated structures (quantified using DINO-SwinL detector; mAP 78.4%).	- PV reduction is associated with exhaustion or selective loss of PV^+^ interneurons, as well as suppressed protein synthesis under persistent oxidative stress. - Extracellular PV likely reflects release from damaged neurons. - Cx43 hyperexpression corresponds to reactive gliosis and structural remodeling of the astrocytic network. - Partial recovery of NeuN^+^ cells suggests transient phenotype loss in a subset of neurons. - A mirror-like pattern emerges: shift from neuronal Ca^2+^ stabilization to glial remodeling.	Sustained PV reduction after TBI and its association with impaired GABAergic inhibition have been reported [36,37,38]. Transient loss of NeuN immunoreactivity without immediate neuronal death has been described [41,42]. Reactive Cx43 upregulation during gliosis has been confirmed in various brain injury models [22]. Regarding the quantitative assessment using computer vision methods: such uncertainty is common in fluorescence microscopy data and contributes to observed detection errors [43,44]. The 78.4% mAP reflects a balance between model capacity and biological annotation noise in post-traumatic samples. Despite limited training data, the model delivered stable, reproducible performance, sometimes surpassing human operators in identifying Cx43 clusters in low-intensity areas. Biologically, this highlights its reliance on intensity thresholds plus morphological/contextual features to average annotation noise and recover patterns—a effect seen in noise-robust learning, implying automated methods can more reliably depict biological structures [45].
Molecular Interactions (in silico, MDS)	- Formation of the most stable PV–Cx43 complex in the presence of two Ca^2+^ ions (minimal Coulombic energy, negative energy drift). - Hydrogen-bond network between N-terminal PV residues and intracellular domains of Cx43. - Ca^2+^-induced conformational rearrangements enhance PV thermodynamic stability.	- Complex is unstable in the absence of Ca^2+^ (positive energy drift). - Interaction weakens under low pH due to protonation of key residues. - Combination of acidosis and Ca^2+^ completely abolishes association.	- Ca^2+^ and pH act as critical regulators of the PV–Cx43 complex. - In the acute phase, Ca^2+^ overload and acidosis may destabilize the interaction and contribute to Cx43 suppression. - In the subacute phase, compensation may occur through PV-independent pathways. - The complex represents a potential therapeutic target.	Neuronal expression of Cx43, including in PV^+^ populations, has been reported [15]. Metabolic alterations, including hypoxia, lactate accumulation, and reduced tissue buffering capacity, lead to acidification of the injured area after TBI [46], with pH values reported to decrease to critically low levels of approximately 6.5 [47]. pH-induced conformational changes in Cx43 and PV have been experimentally described [48,49]. High-affinity Ca^2+^ binding and structural stabilization of PV have been confirmed in biophysical studies [50,51].

## Data Availability

The original contributions presented in this study are included in the article/Supplementary Materials. Further inquiries can be directed to the corresponding author.

## Supplementary Materials

The following supporting information can be downloaded at: https://www.mdpi.com/article/10.3390/molecules31061018/s1, File S1: report_new. Atomic coordinates of the most stable model of the Cx43–PV complex, obtained from molecular dynamics simulations.
